# Synthesis and Characterization of Co(II), Cu(II), and Ni(II) Complexes of 2,2′‐Bipyridine‐4,4′‐Dicarboxamide Ligands: Antibacterial Evaluation Against Resistant Bacteria and Enzyme Inhibition

**DOI:** 10.1002/open.202500097

**Published:** 2025-06-09

**Authors:** Hussein Alchurak, Aseel Almadhoun, İsmail Yılmaz, Raneem Mamoun Ali Nour, Müslüm Kuzu, Hasan Solmaz

**Affiliations:** ^1^ Department of Pharmaceutical Chemistry College of Pharmacy University of Kufa Najaf 540011 Iraq; ^2^ Department of Medical Microbiology The Institute of Graduate Programs Karabük University Karabük 78050 Türkiye; ^3^ Department of Chemistry Faculty of Sciences Karabük University Karabük 78050 Türkiye; ^4^ Department of Food Toxicology The Institute of Graduate Programs Karabük University Karabük 78050 Türkiye; ^5^ Department of Nutrition and Dietetics Faculty of Health Sciences Karabük University Karabük 78050 Türkiye; ^6^ Department of Medical Microbiology Faculty of Medicine Karabük University Karabük 78050 Türkiye

**Keywords:** amide ligands, antibacterial activities, bipyridine, enzyme inhibitions, transition metal complexes

## Abstract

In this study, two new 2,2′‐bipyridine‐4,4′‐dicarboxamide ligands, namely N4,N4′‐bis(pyridin‐2‐ylmethyl)‐[2,2′‐bipyridine]‐4,4′‐dicarboxamide (**L1**) and N4,N4′‐bis(piperidin‐2‐ylmethyl)‐[2,2′‐bipyridine]‐4,4′‐dicarboxamide (**L2**), and their Co(II), Cu(II), and Ni(II) complexes are synthesized and characterized. Elemental analysis, Fourier transform infrared spectroscopy, nuclear magnetic resonance, and mass techniques confirmed the structures of the synthesized compounds. The **L1** structure is also determined by single‐crystal X‐ray diffraction. Antibacterial activities of ligands and metal complexes against resistant strains are investigated. The **L2‐Co** complex demonstrates promising antibacterial qualities against these resistant strains, which is noteworthy. Additionally, enzyme inhibition studies are conducted to assess their potential therapeutic applications. The results show that the **L2‐Ni** complex had the strongest inhibitory effect with IC_50_ values of 1.35 μM for lipase enzyme, 4.33 μM for acetylcholinesterase enzyme, and 6.42 μM for butyrylcholinesterase.

## Graphical abstract

Two new 2,2′‐bipyridine‐4,4′‐dicarboxamide ligands and their Co(II), Cu(II), and Ni(II) complexes are synthesized and characterized. The L2‐Co complex shows strong antibacterial activity against *Staphylococcus aureus*, *Escherichia coli*, and *Pseudomonas aeruginosa*. The L2‐Ni complex displays the most potent enzyme inhibition with IC50 values of 1.35 μM (lipase), 4.33 μM (AChE), and 6.42 μM (BChE).

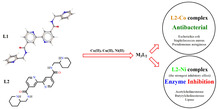

## 1 Introduction

In coordination chemistry, 2,2′‐bipyridine‐bodied ligands have emerged as versatile building blocks for the design of metal complexes, with wide‐ranging applications spanning catalysis, materials science, and medicinal chemistry. We have previously reported the synthesis of metal complexes containing ligands with 2,2′‐bipyridine bodies.^[^ ^1^, ^2^, ^3^, ^4^, ^5^, ^6^, ^7^ ^]^ In addition, our research also includes the synthesis of complexes using ligands derived from 2‐aminomethylpiperidine and 2‐aminomethylpyridine.^[^ ^8^, ^9^ ^]^ In line with our continuing interest in these ligands, we aimed to synthesize symmetric diamide ligands containing a 2,2′‐bipyridine body and prepare metal complexes of these ligands. In particular, numerous studies have highlighted the remarkable biological activity associated with compounds with amide functional groups, underlining the importance of our research.^[^ ^10^, ^11^, ^12^, ^13^ ^]^

Increasing antibacterial resistance necessitates innovative approaches in drug discovery, and new ways are sought for therapeutic agents. Therefore, synthesizing new potential drug substances and determining their biological properties is very important. These complexes formed with synthesized ligands have antibacterial activity and inhibitor potential. Transition metal complexes show diverse biological activities, notably antimicrobial effects. Cobalt(III) complexes with pyridine‐amide ligands have exhibited antibacterial activity against strains like *Pseudomonas*, *Proteus*, and *Escherichia coli*.^[^ ^10^ ^]^ Similarly, a manganese(III) complex with a phenol‐based ligand was effective against various *Vibrio* species, *Aeromonas hydrophila*, and *E. coli*.^[^ ^14^ ^]^

Enzymes responsible for the catalysis of reactions that occur to carry out vital activities in living things also serve as targets in controlling these activities. Due to these features, interventions aimed at regulating their activities can be used for treatment in the treatment processes of pathological conditions. Because in most diseases, abnormalities in certain enzyme activities occur.^[^ ^15^ ^]^ Pancreatic lipase is the primary enzyme involved in metabolizing dietary fats. Excess fat intake causes obesity. It is stated that inhibitors of this enzyme can be used in treating obesity and related diseases because they prevent absorption from the small intestine. Considering that the only antiobesity drug approved by the Food and Drug Administration (FDA) is a pancreatic lipase inhibitor (orlistat), the importance of discovering new and potent inhibitors for the enzyme is better understood.^[^ ^16^ ^]^ It has been reported that there will be a need to find new therapeutics due to the expected increase in Alzheimer's disease (AD) cases. Cholinergic hypothesis one of the most important approaches used in the treatment of AD is the cholinergic hypothesis, and both acetylcholinesterase (AChE) and butyrylcholinesterase (BChE) enzymes play a significant role in the cholinergic system. It has been reported that the AChE enzyme is used as a target in the treatment of AD to stop the progression of the disease, and its inhibitors have clinical applications.^[^ ^17^ ^]^

This study reveals the synthesis, characterization, potential antibacterial activities, and enzyme inhibition properties of two 2,2′‐bipyridine‐4,4′‐dicarboxamide ligands and their Co(II), Cu(II), and Ni(II) complexes. In this context, our research aims to shed light on the biological activities of the synthesized metal complexes, their antibacterial activities against resistant strains, and their potential as enzyme inhibitors.

## 2 Experimental Section

### 2.1 Materials and Instruments

All of the chemicals were purchased from commercial sources and utilized directly. 4,4′‐dimethyl‐2,2′‐bipyridine, 2‐(aminomethyl)pyridine from Acros; nickel(II)‐acetate, 2‐(aminomethyl)piperidine, benzene from Sigma‐Aldrich; NaOH from Carlo Erba; petroleum ether, K_2_Cr_2_O_7_ from Riedel‐de Haen, copper(II) acetate monohydrate, thionyl chloride, H_2_SO_4_, dichloromethane, methanol, ethanol, cobalt(II)‐acetate were purchased from Merck. The compounds were analyzed using FT‐IR (Nicolet iS5, Thermo Scientific) coupled with the attenuated total reflection (ATR) technique (iD7, Thermo Scientific). The elemental analyses of ligands were carried out using an LECO Truspec Micro CHN microanalysis apparatus with LECO Truspec Micro. The ^1^H‐NMR and ^13^C‐NMR spectra of ligands were recorded on an Agilent 600 MHz spectrometer using DMSO‐*d*_*6*_ solvent. MS analysis of ligands was carried out on a Thermo Scientific TSQ Quantum Access MAX triple quadrupole mass spectrometer. Mass spectra of metal complexes were performed on a Bruker Microflex LT MALDI‐TOF MS. Melting points were measured with a Thermo Fisher Scientific IA9100 apparatus. The compound (L1) suitable for single crystal X‐ray diffraction was obtained by slowly evaporating its solution in methanol at room temperature. Details of data collection and crystal structure determinations are given in **Table** **1**. Crystallographic data for the structural analysis of 1 have been deposited with the Cambridge Crystallographic Data Centre, CCDC 2320722. The data was collected on a D8‐QUEST diffractometer equipped with graphite‐monochromatic Mo‐Kα radiation at 296 K. Using Olex2,^[^ ^18^ ^]^ the structure was solved with the olex2.solve^[^ ^19^ ^]^ structure solution program using Charge Flipping and refined with the olex2.refine^[^ ^19^ ^]^ refinement package using Gauss–Newton minimization and with SHELXL.^[^ ^20^ ^]^ Molecular diagrams were generated using OLEX2^[^ ^18^ ^]^ and MERCURY.^[^ ^21^ ^]^

**Table 1:** Crystal data and structure refinement for L1.

Empirical formula	C_24_H_20_N_6_O_2_
Formula weight	424.465
Temperature [K]	296
Crystal system	Monoclinic
Space group	P2_1_/c
*a* [Å]	15.040(2)
*b* [Å]	7.8397(12)
*c* [Å]	9.2744(12)
*α* [°]	90
*β* [°]	103.203(5)
*γ* [°]	90
Volume [Å^3^]	1064.6(3)
*Z*	2
ρ_calc_ [g cm^−3^]	1.324
*μ* [mm^−1^]	0.088
F(000)	444.3
Crystal size [mm^3^]	0.11 × 0.07 × 0.05
Radiation	Mo Kα (*λ* = 0.71073)
2Θ range for data collection [°]	5.56 to 51.98
Index ranges	−20 ≤ *h* ≤ 20, −10 ≤ *k* ≤ 10, −12 ≤ *l* ≤ 12
Reflections collected	34 570
Independent reflections	2086 [*R*_int_ = 0.0883, *R*_sigma_ = 0.0639]
Data/restraints/parameters	2086/0/146
Goodness‐of‐fit on *F* ^2^	1.086
Final *R* indexes [*I* > = 2*σ* (*I*)]	*R*_1_ = 0.0478, *wR*_2_ = 0.1118
Final *R* indexes [all data]	*R*_1_ = 0.1075, *wR*_2_ = 0.1464
Largest diff. peak/hole [e Å^−3^]	0.31/−0.30

### 2.2 Synthesis of the Compounds

#### 2.2.1 General Synthetic Procedure for Ligands

Syntheses of the ligands were carried out by modifying literature methods.^[^ ^22^ ^]^ 4,4′‐dimethyl‐2,2′‐bipyridine (5 g, 27 mmol) was dissolved in 125 mL concentrated H_2_SO_4_ at 70 °C. 24 g (82 mmol) K_2_Cr_2_O_7_ was added slowly, and a dark green solution was formed. The mixture was stirred for 5 h and poured into 800 mL of water‐ice. The light‐yellow precipitate was filtered by vacuum filtration and washed with plenty of water. The resulting solid was added to 150 mL of 50% HNO_3_ solution and boiled for 4 h. The mixture was poured back into 800 mL of water‐ice. The white precipitate formed was separated by vacuum filtration, washed with plenty of water, and dried in open air. The obtained 2,2′‐bipyridine‐4,4′‐dicarboxylic acid (2 g, 8.2 mmol) was boiled in a mixture of 20 mL SOCl_2_ and 10 mL benzene for 24 h. After the excess SOCl_2_ evaporated, the reaction mixture was washed with petroleum ether and dried. Then, acid chloride (1 g, 3.6 mmol) dissolved in 40 mL dichloromethane and amine (7.2 mmol) dissolved in 20 mL dichloromethane were slowly mixed at 0 °C, and 20 mL 0.5 M NaOH solution was added. The mixture was stirred at 0 °C for 5 h and at room temperature for 19 h. The white precipitate formed was filtered and washed with plenty of water. After drying, it was crystallized in methanol.

#### 2.2.2 4,4′‐Dicarboxy‐2,2′‐Bipyridine

FT‐IR spectrum [ATR/cm^−1^]: 3743, 3111, 3057, 2358, 1868, 1709, 1603, 1561, 1457, 1365, 1268, 1235, 1194, 1139, 1067, 1016, 913, 866, 820, 764, 720, 680. ^1^H NMR (600 MHz, DMSO‐*d*_6_) δ 13.77 (s, 1H, COOH), 8.90 (d, *J* = 4.9 Hz, 1H, bpy6,6′), 8.83 (s, 1H, bpy3,3′), 7.90 (d, *J* = 4.5 Hz, 1H, bpy5,5′). ESI‐MS (m/z): 245.40 (Calc. for C_12_H_8_N_2_O_4_ 244.21). Yield = 85%, m.p. 320 °C (decomposed).

#### 2.2.3 4,4′‐Bis(chlorocarbonyl)‐2,2′‐Bipyridine

FT‐IR spectrum [ATR/cm^−1^]: 3066, 1751, 1586, 1550, 1452, 1356, 1247, 1193, 1065, 905, 855, 734, 699, 673. Yield = 87%, m.p. 358 °C.

#### 2.2.4 L1

FT‐IR spectrum [ATR/cm^−1^]: 3301, 3061, 2934, 1637, 1590, 1571, 1539, 1470, 1435, 1419, 1388, 1355, 1312, 1252, 1166, 1146, 1101, 1073, 1050, 994, 913, 865, 817, 769, 753, 733, 700, 656, 630, 607, 549, 476, 434. ^1^H NMR (600 MHz, DMSO‐*d*_6_) δ 9.58 (t, *J* = 5.9 Hz, 1H, NH), 8.88 (d, *J* = 7.9 Hz, 2H), 8.52 (d, *J* = 5.1 Hz, 1H), 7.92 (d, *J* = 5.1 Hz, 1H), 7.76 (t, *J* = 7.8 Hz, 1H), 7.36 (d, *J* = 8.1 Hz, 1H), 7.27 (dd, *J* = 7.6, 4.7 Hz, 1H), 4.62 (d, *J* = 5.9 Hz, 2H, CH_2_). ^13^C NMR and DEPT (151 MHz, DMSO) δ 165.26(C), 158.67(C), 156.04(C), 150.57(CH), 149.37(CH), 143.07(C), 137.21(CH), 122.65(CH), 122.45(CH), 121.57(CH), 118.79(CH), 45.31(CH_2_). Elemental analysis calcd for C_24_H_20_N_6_O_2_·0,5H_2_O (%): C, 66.50; H, 4.88; N, 19.39; found: C, 66.47; H, 4.81; N, 19.17. ESI‐MS (m/z): 425.10 (Calc. for C_24_H_20_N_6_O_2_ 424.46). Yield = 88%, m.p. 230 °C.

#### 2.2.5 L2

FT‐IR spectrum [ATR/cm^−1^]: 3308, 3063, 2931, 2857, 1641, 1593, 1545, 1410, 1355, 1311, 1243, 1204, 1168, 1139, 1121, 1075, 1053, 1020, 999, 906, 856, 799, 759, 683, 662, 640, 565, 513, 445, 429, 414, 409, 403. ^1^H NMR (600 MHz, DMSO‐*d*_6_) δ 8.87 (t, *J* = 6.4 Hz, 1H, NH), 8.84 (d, *J* = 5.4 Hz, 1H, bpy6,6′), 8.77 (s, 1H, bpy3,3′), 7.84 (d, *J* = 6.2 Hz, 1H, bpy5,5′), 3.26–3.23 (m, 1H), 3.20 (dt, *J* = 13.4, 6,6 Hz, 1H), 2.92 (d, *J* = 11.9 Hz, 1H), 2.65 (q, *J* = 7.8 Hz, 1H), 1.71 (d, *J* = 12.0 Hz, 1H), 1.60 (d, *J* = 12.6 Hz, 1H), 1.48 (d, *J* = 10.3 Hz, 1H), 1.27 (q, *J* = 11.4 Hz, 2H), 1.03 (q, *J* = 11.9 Hz, 1H). ^13^C NMR and DEPT (151 MHz, DMSO) δ 165.26(C), 155.94(C), 150.38(CH), 143.43(C), 122.45(CH), 118.77(CH), 56.20(CH), 46.51(CH_2_), 45.73(CH_2_), 30.55(CH_2_), 26.31(CH_2_), 24.57(CH_2_). Elemental analysis calcd for C_24_H_32_N_6_O_2_·1,5H_2_O (%): C, 62.18; H, 7.61; N, 18.13; found: 62.49; H, 7.16; N, 17.84. ESI‐MS (m/z): **436.79** (Calc. for C_24_H_32_N_6_O_2_ 436.56). Yield = 58%, m.p. 237 °C.

### 2.3 General Synthetic Procedure for Complexes

1 equv of metal acetate salt (Cu(II), Co(II), and Ni(II)) dissolved in 20 mL of methanol was added slowly to 1 equv of ligand dissolved in 30 mL of methanol at room temperature. The resulting‐colored solution was stirred for 1 h. After completely removing the methanol in the evaporator, the substances were dissolved in 30 mL of water, filtered, and crystallized in the open air. Shiny‐crystalline substances were dried in a desiccator. Unfortunately, suitable single crystals of these complexes for X‐ray analysis could not be obtained.

#### 2.3.1 L1‐Co Complex (1)

Color: Dark orange. FT‐IR spectrum [ATR/cm^−1^]: 3228, 3063, 2970, 1656, 1592, 1537, 1477, 1397, 1319, 1281, 1234, 1218, 1151, 1127, 1099, 1047, 1002, 865, 756, 692, 654, 607, 570, 527, 496, 431, 421, 416, 405. MALDI‐TOF MS (m/z): [LH]^+^: 425.329; [CoL(OAc)_2_(H_2_O)_2_]^+^: 637.364; [CoL_2_]^+^: 907.591; [Co_2_L_2_(OAc)_2_(H_2_O)_2_‐H]^+^: 1119.031; [Co_3_L_2_(OAc)_4_(H_2_O)_4_‐2H]^+^: 1330.900.

#### 2.3.2 L1‐Cu Complex (2)

Color: Dark green. FT‐IR spectrum [ATR/cm^−1^]: 3248, 3065, 2935, 1654, 1542, 1476, 1398, 1320, 1285, 1236, 1202, 1169, 1151, 1100, 1047, 1003, 909, 861, 755, 693, 631, 611, 527, 488, 429, 414, 402. MALDI‐TOF MS (m/z): [LH]^+^: 425.144; [CuL‐H]^+^: 489.699; [CuL_2_]^+^: 912.500; [Cu_2_L_2_ + 2H]^+^: 978.611; [Cu_3_L_2_(H_2_O)‐2H + 0.5 H_2_O]^+^: 1069.266.

#### 2.3.3 L1‐Ni Complex (3)

Color: Green. FT‐IR spectrum [ATR/cm^−1^]: 3228, 3067, 2969, 1657, 1592, 1533, 1479, 1399, 1321, 1283, 1234, 1218, 1201, 1171, 1153, 1129, 1101, 1046, 1022, 1003, 941, 900, 864, 756, 718, 692, 661, 614, 527, 489, 420, 402. MALDI‐TOF MS (m/z): [LH]^+^: 425.390; [NiL‐H]^+^: 481.533; [NiL_2_ + H]^+^: 909.191; [Ni_2_L_2_]^+^: 966.020; [Ni_3_L_2_‐2H]^+^: 1023.304.

#### 2.3.4 L2‐Co Complex (4)

Color: Dark orange. FT‐IR spectrum [ATR/cm^−1^]: 3235, 2939, 2862, 1657, 1550, 1445, 1401, 1335, 1276, 1234, 1204, 1170, 1079, 1055, 1039, 966, 922, 861, 754, 648, 614, 516, 470, 452, 429, 414, 408. MALDI‐TOF MS (m/z): [LH + 0.5 H_2_O]^+^: 446.842; [Co_3_L(OAc)_2_(H_2_O)_3_‐3 H]^+^: 782.707; [Co_3_L_2_(H_2_O)_3_+H]^+^: 1104.564.

#### 2.3.5 L2‐Cu Complex (5)

Color: Green. FT‐IR spectrum [ATR/cm^−1^]: 3248, 3110, 2940, 2859, 1651, 1553, 1478, 1401, 1340, 1286, 1235, 1160, 1126, 1088, 1040, 955, 932, 857, 757, 647, 616, 501, 419, 413, 408, 402. MALDI‐TOF MS (m/z): [LH]^+^: 437.530; [CuL_2_ + H]^+^: 938.468; [Cu_3_L_2_(OAc)_5_(H_2_O)_4_‐H]^+^: 1430.345.

#### 2.3.6 L2‐Ni Complex (6)

Color: Light green. FT‐IR spectrum [ATR/cm^−1^]: 3240, 3080, 2936, 2861, 1656, 1549, 1476, 1403, 1340, 1280, 1234, 1203, 1167, 1134, 1084, 1046, 1018, 946, 927, 868, 853, 836, 759, 650, 617, 467, 407. MALDI‐TOF MS (m/z): [LH + 0.5 H_2_O]^+^: 447.758; [NiL(OAc)(H_2_O)]^+^: 572.907; [NiL_2_ + 2H]^+^: 933.700; [Ni_2_L_2_(OAc)_4_‐H + 0.5H_2_O]^+^: 1234.784; [Ni_3_L_2_(OAc)_4_(H_2_O)‐3 H + 0.5 H_2_O]^+^: 1309.820.

### 2.4 In Vitro Antibacterial Activity

#### 2.4.1 Bacterial Strain

The standard bacterial strains used in antibacterial testing, including *Staphylococcus aureus* (ATCC 29213), *Escherichia coli* (ATCC 70028), and *Pseudomonas aeruginosa* (ATCC 27853), were streaked onto blood agar plates to obtain single colonies from stock cultures. The plates were then incubated overnight at 37 °C. Single colonies that formed were subsequently inoculated into Muller Hinton Broth (MHB). These cultures were incubated at 37 °C for 18–24 h. Following incubation, the bacterial cultures were adjusted to a density of 0.5 McFarland standard to prepare bacterial suspensions for testing. Initially, compounds L1‐Co, L1‐Cu, L1‐Ni, L2‐Co, L2‐Cu, and L2‐Ni were tested using the microdilution technique by testing all the compounds at a concentration of 2000 ppm, where 1 mL of bacteria suspension was added to each tube containing a different compound. The contents were mixed well to ensure the bacteria and compounds were evenly distributed. After the 24‐h incubation period, a sample was cultured on a blood agar plate to determine bacterial growth. The compounds that showed an effect and prevented bacterial growth were also tested using a minimum inhibitory concentration test (MIC). The optical density (OD) was measured at 600 nm, with a target OD of 0.063, using a spectrophotometer to ensure accurate bacterial concentration.

#### 2.4.2 Dilution of Complexes

For L2‐Co, a stock concentration of 2 mg mL^−1^ was prepared in aqueous solution and diluted with distilled water to obtain final concentrations of 1000 and 500 ppm. One milliliter of sterile distilled water was transferred into two separate tubes to prepare these different concentrations. In the first tube, 1 mL of the synthesized compound was added and mixed with a pipette, resulting in a 1000 ppm dilution. To achieve a 500 ppm dilution in the second tube, 1 mL was taken from the first tube and transferred to the second tube. Consequently, 2000 (stock solution), 1000, and 500 ppm mL^−1^ were prepared for testing.

#### 2.4.3 Minimum Inhibition Concentration

For each standard bacterial strain, the microplate was prepared with the following controls and test conditions: a positive control (culture adjusted to McFarland 0.5), a negative control with broth (MHB), and test wells with dilutions of cobalt compound stock solution (2000, 1000, and 500 ppm mL^−1^). Specifically, 200 μL of McFarland 0.5 adjusted culture was added to the positive control well, and 200 μL of MHB was added to the negative control well. In the three test wells, 100 μL of McFarland 0.5 adjusted culture was added. Subsequently, 100 μL of the cobalt compound solutions at 2000, 1000, and 500 ppm mL^−1^ were added to the respective test wells. The 96‐well microplate was placed into the spectrophotometer (Thermo), and the first reading was recorded as the zero‐hour baseline. Subsequent readings were taken and recorded at 2, 4, 6, 24, 30, and 48 h. The MIC was determined to be the lowest concentration of the compound, which completely inhibited visible bacterial growth.

### 2.5 In Vitro Inhibition Assay

In cholinesterase activity measurements, the method reported by Karagecili and coworkers was used with minor modifications.^[^ ^23^ ^]^ Here, acetylchoiniodate was used as a substrate for both enzymes. Lipase enzyme activity measurement was performed according to the method reported by Bulut.^[^ ^24^ ^]^ In inhibition studies, measurements were made at at least five different concentrations of the molecules synthesized. %activity values were calculated by accepting enzyme activity measurements without inhibitor as 100%. Then, molecule concentrations were plotted against Activity% values. IC_50_ (inhibitor concentration providing 50% inhibition) values were calculated for molecules that caused more than 50% inhibition from the resulting graphic equation.^[^ ^25^ ^]^

### 2.6 Docking Studies

The Maestro 12.5 program of Schrödinger Molecular Modeling Suite was used in the study. The software uses two important programs when performing operations. These are Emodel and GlideScore. EmodelScore determines the ideal posture of the ligand. GlideScore gives ligand binding affinity results. Three‐dimensional crystal structures of proteins were obtained from the RCSB Protein Data Bank. This study transferred the structures specified with PDB codes 4TVK for AChE enzyme, 4BDS for BChE, and 1LPB for pancreatic lipase to Maestro Release 2023‐1. Protein preparation for the docking study was performed using Schrödinger's Protein Preparation Wizard. Maestro's Receptor Grid Generation Application identified the prepared enzyme's active site. The molecular structures of the ligands used in the synthesis of metal complexes were drawn in the ChemDraw 18.0 application, and the molecular structures of the standard inhibitors of the enzymes were taken from the www.pubchem.ncbi.nlm.nih.gov page and uploaded to the program. These molecules were made ready for docking with Maestro's Ligprep application. Glide/XP was used to dock all compounds into the abovementioned enzymes. The conformations of the ligands with the lowest binding free energy were evaluated. Interaction diagrams and connection patterns were obtained with Maestro Release 2023‐1.

## 3 Results and Discussion

### 3.1 Synthesis and Characterization

**Scheme 1:**
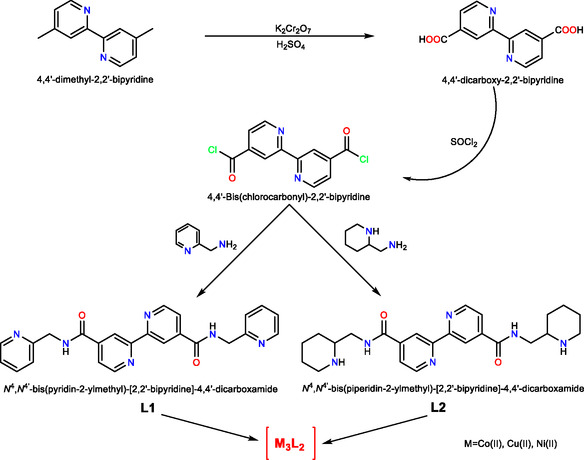
Synthetic routes for the ligands and their corresponding complexes.

The L1 and L2 ligands were synthesized from the commercially available 4,4′‐dimethyl‐2,2′‐bipyridine compound by following the steps of carboxylation, acylation, and condensation of acyl chloride with the corresponding amine, as shown in **Scheme** **1**.^[^ ^22^ ^]^ The metal complexes were obtained by stirring the ligand and metal salts in methanol at a ratio of 1:1 at room temperature. The ligands were characterized by FT‐IR(ATR), ^1^H‐NMR, ^13^C‐NMR‐DEPT, ESI‐MS, and elemental analysis techniques; metal complexes were characterized by FT‐IR and MALDI TOF‐MS techniques. All analyses of the compounds are listed in the experiments section. The characteristic C=O stretching frequencies were observed at 1709 cm^−1^ for 4,4′‐dicarboxy‐2,2′‐bipyridine, 1751 cm^−1^ for 4,4′‐Bis(chlorocarbonyl)‐2,2′‐bipyridine, 1637 cm^−1^ for L1, and 1641 cm^−1^ for L2 and between 1651 and 1657 cm^−1^ for complexes. As a result of the complexation, the C=O_amide_ peaks showed blue‐shifting between 10 and 20 cm^−1^. The characteristic N—H_amide_ stretching frequencies were observed at 3301 cm^−1^ for L1, 3308 cm^−1^ for L2, and 3228‐3248 cm^−1^ for complexes. As a result of the complexation, the N—H_amide_ peaks showed red‐shifting between 53 and 73 cm^−1^. The ^1^H‐NMR spectrum of 4,4′‐dicarboxy‐2,2′‐bipyridine is similar to previously reported.^[^ ^26^ ^]^

**Figure 1:**
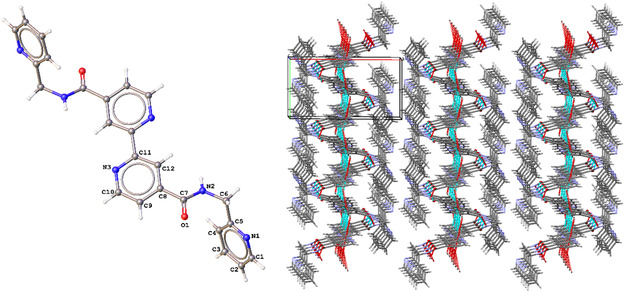
Partially labeled molecular structure and molecular packing view (3 × 3 × 3) for **L1**.

The NMR technique determined the protons and carbon atoms of the ligands. In addition, the hydrogen numbers in carbon atoms were determined by a modern NMR technique called DEPT. The symmetrical 16 protons of the L2 ligand are also compatible with the molecular structure. Amide NH protons were observed as triplet at 9.58 ppm in L1 and 8.87 ppm in L2. In the L1 ligand, aliphatic CH_2_ protons were observed as a doublet at 4.62 ppm, and the coupling value between aliphatic CH_2_ and amide NH protons was determined as 5.9 Hz. The aliphatic CH_2_ proton peaks of L2 neighboring the amide NH were observed at 3.25 and 3.20 ppm. The symmetrical 12 carbon atoms of the L1 and L2 ligands have also been fully determined. ESI‐MS and elemental analyses of ligands are consistent with their structures. The MALDI TOF‐MS analyses show that the complex structures are in the structure [M_3_L_2_]. The details of the crystal data collection and refinement of L1 are summarized in Table 1. The bond distances and angles of the ligand are collected in **Table** **2**. The partially labeled molecular structure and molecular packaging view for L1 are shown in **Figure** **1**. The L1 ligand crystallizes with space group P21/c in the monoclinic crystal system. The unit cell parameters were determined to be *a* = 15.040(2) Å, *b* = 7.8397(12) Å, *c* = 9.2744(12) Å, *α* = 90°, *β* = 103.203(5)°, *γ* = 90°, *V* = 1064.6(3) Å^3^, *Z* = 2. In the crystal structure of the **L1**, the crystal packing is stabilized by intermolecular hydrogen bonds, CH···π, C=O···N and π··· π interactions, forming a 3D network (see Figure 1).

**Table 2:** Bond lengths and bond angles for L1.

Atom	Atom	Length [Å]	Atom	Atom	Length [Å]	Atom	Atom	Atom	Angle [°]	Atom	Atom	Atom	Angle [°]
C1	C2	1.348(5)	C7	N2	1.329(3)	N1	C1	C2	124.5(3)	C9	C8	C7	118.55(18)
C1	N1	1.332(4)	C7	O1	1.233(2)	C3	C2	C1	118.4(4)	C12	C8	C7	123.72(19)
C2	C3	1.354(5)	C8	C9	1.386(3)	C4	C3	C2	118.7(3)	C12	C8	C9	117.73(19)
C3	C4	1.382(5)	C8	C12	1.383(3)	C5	C4	C3	119.2(3)	C10	C9	C8	119.21(19)
C4	C5	1.358(4)	C9	C10	1.373(3)	C6	C5	C4	123.1(2)	N3	C10	C9	124.2(2)
C5	C6	1.501(3)	C10	N3	1.333(3)	N1	C5	C4	122.5(2)	N3	C11	C12	122.61(18)
C5	N1	1.333(3)	C11	C11^1^	1.486(4)	N1	C5	C6	114.5(2)	C11	C12	C8	119.45(19)
C6	N2	1.451(3)	C11	C12	1.393(3)	N2	C6	C5	114.28(18)	C5	N1	C1	116.6(3)
C7	C8	1.497(3)	C11	N3	1.348(3)	N2	C7	C8	116.95(17)	C7	N2	C6	120.68(17)
–		–	–	–	–	O1	C7	C8	120.43(19)	C11	N3	C10	116.81(19)
^1^1−*X*,2−*Y*,−*Z*		–	–	–	–	O1	C7	N2	122.63(19)	–	–	–	–

### 3.2 Antimicrobial Activity Data

#### 3.2.1 Antimicrobial Activity Data by Macro Dilution

The results for L1, L2, L1‐Co, L1‐Cu, L1‐Ni, L2‐Co, L2‐Cu, and L2‐Ni are presented in **Table** **3**. Agar plates were inspected after the 24‐h incubation period to assess bacterial growth. In dishes where no bacterial growth was observed, the concentration of the substance is considered sufficient to inhibit bacterial growth.

**Table 3:** Antimicrobial activity results by macro dilution method.

	*Staphylococcus aureus*	*Escherichia coli*	*Pseudomonas aeruginosa*
Positive control	+	+	+
L1	+	+	+
L2	+	+	+
L1‐Co	+	+	+
L1‐Cu	+	+	+
L1‐Ni	+	+	+
L2‐Co	–	–	–
L2‐Cu	+	+	+
L2‐Ni	+	+	+

#### 3.2.2 Antimicrobial Activity Data by MIC

Bacterial growth was quantified by measuring absorbance at 600 nm using a spectrophotometer. Data was analyzed in Excel to calculate MIC values. All assays included positive controls (McFarland 0.5 adjusted culture) and negative controls (DMSO), as shown in **Table** **4**. Table 4 presents spectrophotometric measurements taken at various intervals for bacterial species exposed to L2‐Co alongside positive and negative controls.

**Table 4:** Spectrophotometry results were taken at different time intervals for bacterial strains containing L2‐Co.

	0 h	2 h	4 h	6 h	24 h	30 h	48 h
Negative control	0.067	0.066	0.065	0.064	0.064	0.064	0.064
Positive control *Escherichia coli*	0.072	0.072	0.074	0.082	0.259	0.289	0.407
500 ppm **L2‐Co** *Escherichia coli*	0.061	0.064	0.065	0.067	0.068	0.069	0.074
1000 ppm **L2‐Co** *Escherichia coli*	0.070	0.075	0.077	0.078	0.080	0.085	0.099
2000 ppm **L2‐Co** *Escherichia coli*	0.086	0.091	0.093	0.093	0.100	0.099	0.101
Positive control *Pseudomonas aeruginosa*	0.061	0.065	0.075	0.093	0.384	0.439	0.647
500 ppm **L2‐Co** *Pseudomonas aeruginosa*	0.059	0.060	0.062	0.063	0.064	0.064	0.077
1000 ppm **L2‐Co** *Pseudomonas aeruginosa*	0.068	0.072	0.073	0.075	0.075	0.077	0.096
2000 ppm **L2‐Co** *Pseudomonas aeruginosa*	0.086	0.090	0.093	0.095	0.099	0.099	0.101
Positive control *Staphyloccocus aureus*	0.063	0.064	0.067	0.075	0.267	0.300	0.411
500 ppm **L2‐Co** *Staphyloccocus aureus*	0.064	0.065	0.066	0.067	0.068	0.068	0.072
1000 ppm **L2‐Co** *Staphyloccocus aureus*	0.070	0.073	0.074	0.075	0.076	0.077	0.089
2000 ppm **L2‐Co** *Staphyloccocus aureus*	0.093	0.097	0.099	0.100	0.102	0.102	0.104

The initial results for all three bacterial species indicated that most compounds did not have inhibitory effects, as bacterial growth was observed similarly to the positive control and based on the growth patterns in Table 3, *Escherichia coli*, *Pseudomonas aeruginosa*, and *Staphylococcus aureus* continued to grow in the presence of L1‐Co, L1‐Cu, L1‐Ni, L2‐Cu, and L2‐Ni at various concentrations, showing that these compounds did not hinder bacterial growth.

In contrast, L2‐Co exhibited significant antimicrobial activity across all three bacterial species. As shown in Table 3 and 4, even at the lowest concentration tested (500 ppm), L2‐Co effectively reduced bacterial growth in *Escherichia coli*, *Pseudomonas aeruginosa*, and *Staphylococcus aureus*. The antibacterial effect of L2‐Co is further demonstrated by the values in Table 4, suggesting that its efficacy may be due to its hydrophobic nature, which could damage bacterial cell walls.^[^ ^27^ ^]^

However, the exact mechanisms by which L2‐Co may impact human health, based on its physical and chemical properties, remain unclear and require further investigation.

#### 3.2.3 Inhibition Results

**Figure 2:**
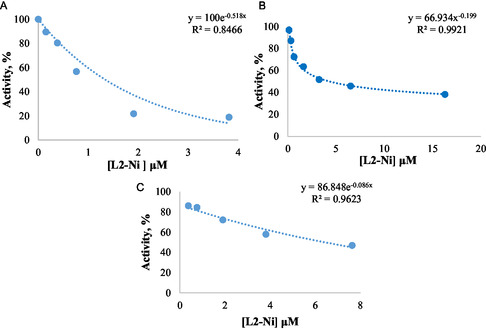
% Activity–concentration graphs for the complexes with the strongest inhibitory effect. A) For pancreatic lipase, B) AChE, and C) BChE.

The results obtained in the inhibition studies are summarized in **Table** **5**. Accordingly, L2‐Ni showed the strongest inhibition effect for pancreatic lipase and AChE enzyme and L2‐Cu for BChE enzyme (Table 5). However, in the concentration ranges studied, L1 did not show a linear effect on lipase, L1‐Co on lipase and AChE enzyme, and L1‐Ni on AChE. In their study, Serbest et al. reported that the 2,2′‐Bipyridine‐Ni complex inhibited the AChE enzyme with a value of 14 μM IC_50_.^[^ ^28^ ^]^ Our study determined that the L2‐Ni complex inhibited the enzyme with an IC_50_ value of 4.33 μM (**Figure** **2** **)**.

**Table 5:** For ligands and complexes, studied concentration ranges and IC_50_ values.

	Concentration range [μM]			IC50 values [μM]		
	For Lipase	For AChE	For BChE	Lipase	AChE	BChE
**L1**	1.89–377.12	7.86–314.27	3.77–188.56	–	173.3	68.07
**L2**	1.83–91.63	1.53–76.35	1.83–183.25	24.08	10.21	8.834
**L1‐Cu**	2.34–46.77	0.19–46.77	0.19–46.77	26.66	23.9	21.66
**L1‐Co**	0.75–37.59	0.15–37.59	0.15–37.59	–	–	34.66
**L1‐Ni**	0.98–24.44	0.20–9.78	0.49–48.88	10.50	–	24.76
**L2‐Cu**	0.11–13.99	0.14–17.48	0.70–34.97	7.89	5.545	6.48
**L2‐Co**	0.14–18.12	0.18–45.29	0.18–45.29	4.74	24.76	22.36
**L2‐Ni**	0.15–3.82	0.13–16.29	0.38–7.64	1.34	4.33	6.42

#### 3.2.4 Docking Results

**Figure 3:**
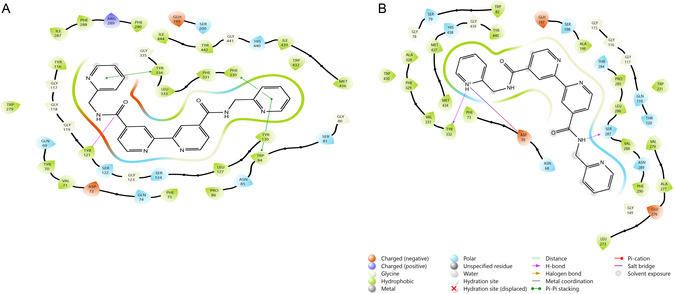
2D image of the interaction of enzymes with the L1 ligand. A) AChE and B) BChE.

Within the scope of the study, a docking study was planned to reveal the interaction mechanisms of the metal complexes whose effects on enzyme activities were examined. However, due to the large molecular structures of the metal complexes, ligand preparation could not be done using the docking program. For this reason, docking studies were carried out only for the ligands. Hydrophobic interaction occurred between the L1 and AChE residues TYR70, VAL71, PHE75, TRP84, PRO86, TYR116, TYR121, LEU127, TYR130, TRP279, ILE287, PHE288, PHE290, PHE330, PHE331, LEU333, TYR334, TRP432, MET436, ILE439, TYR442, and ILE444. Charged (negative) interaction occurred with residues ASP72 and GLH199; positive interaction occurred with residue ARG289. Polar interaction occurred with residues GLN69, GLN74, SER81, ASN85, SER122, SER124, SER200, and HIS440. H‐bond occurred with TYR121. Pi‐Pi stacking interaction occurred with residues TRP84, TYR121, and TYR334. It was observed that the docking score of the ligand–enzyme interaction was −11.350, which was better compared to the value of tacrine (−9.670, **Table** **6**), the standard inhibitor of the enzyme (**Figure** **3**).

**Table 6:** Docking scores (kcal) were obtained for ligands and standard inhibitors of enzymes.

		Docking score	XP GScore	Glide emodel
AChE	L1	−9.205	−9.205	−113.854
	L2	−11.350	−11.350	−98.266
	Tacrine	−9.670	−9.671	−46.072
BChE	L1	−5.809	−8.029	−92.666
	L2	−7.962	−7.963	−76.065
	Tacrine	−6.913	−6.913	−42.707
Lipase	L1	–	–	–
	L2	−3.147	−3.148	−42.248
	Methoxy phosphinic acid	−0.863	−0.863	−20.917

Hydrophobic interaction occurred between the L1 and BChE residues PHE73, TRP82, ALA199, TRP231, LEU273, ALA277, VAL279, PRO285, LEU286, VAL288, PHE290, ALA328, PHE329, VAL331, TYR332, TRP430, MET434, MET437, and TYR440. Charged (negative) interaction occurred with residues ASP70, GLH197, and GLU276. Polar interaction occurred with residues ASN68, SER79, GLN119, THR120, SER198, THR284, SER287, ASN289, and HIS438. H‐bond occurred with SER287 and TYR332. Salt bridge interaction occurred with residue ASP70. It was observed that the docking score of the ligand–enzyme interaction was −5.809, which was lower than the value of tacrine (−6.913), the standard inhibitor of the enzyme.

**Figure 4:**
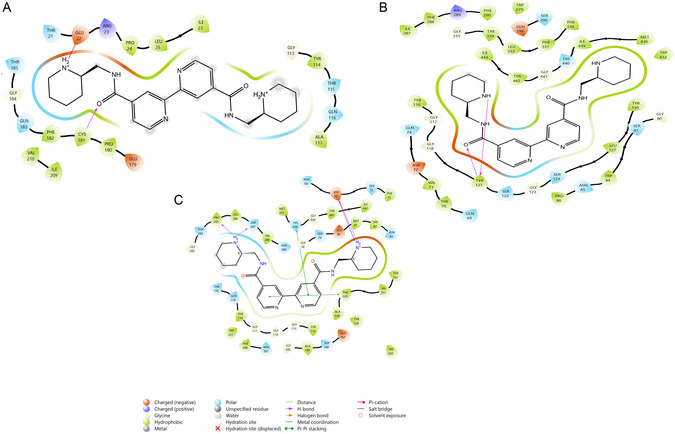
2D image of the interaction of enzymes with the L2 ligand. A) Pancreatic lipase, B) AChE, C) BChE.

Hydrophobic interaction occurred between the L2 and pancreatic lipase residues PRO24, LEU25, ILE27, TYR114, ALA117, PRO180, CYS181, PHE182, ILE209, and VAL210. Charged (negative) interaction occurred with residues GLU22 and GLU179, and positive interaction occurred with residue ARG23. Polar interaction occurred with residues THR21, THR115, GLN116, GLN183, and THR185. H‐bond occurred with CYS181. Salt bridge interaction occurred with residue GLU22. It was observed that the docking score of the ligand–enzyme interaction was −3.147, which was better than the value of methoxy(undecyl)phosphinic acid (−0.863), the standard inhibitor of the enzyme. Hydrophobic interaction occurred between the L2 and AChE residues TYR70, VAL71, TRP84, PTO86, TYR116, TYR121, LEU127, TYR130, TRP279, ILE287, PHE288, PHE290, PHE330, PHE331, LEU333, TYR334, TRP432, MET436, ILE439, TYR442, and ILE444. Charged (negative) interaction occurred with residues ASP72 and GLH199, and positive interaction occurred with residue ARG289. Polar interaction occurred with residues GLN69, GLN74, SER81, ASN85, SER122, SER124, SER200, and HIS440. H‐bond occurred with residue TYR121. It was observed that the docking score of the ligand–enzyme interaction was −11.350, which was better compared to the value of tacrine (−9.670), the standard inhibitor of the enzyme. Hydrophobic interaction occurred between the L2 and BChE residues PHE73, MET81, TRP82, TYR114, PHE118, TYR128, ALA199, TRP231, PRO285, LEU286, VAL288, ALA328, PHE329, VAL331, TYR332, PHE398, TRP430, MET437, TYR440, and ILE442. Charged (negative) interaction occurred with residues ASP70, GLU80, and GLH197. Polar interaction occurred with residues ASN68, SER72, SER79, ASN83, GLN119, THR120, SER198, THR284, SER287, ASN289, ASN397, and HIS438. Pi‐Pi stacking occurred with residues PHE329 and HIS438. Salt bridge occurred with residue ASP70. It was observed that the docking score of the ligand–enzyme interaction was −7.962, which was better compared to the value of tacrine (−6.913), the standard inhibitor of the enzyme (**Figure** **4**).

## 4 Conclusion

In this work two new 2,2′‐bipyridine‐4,4′‐dicarboxamide ligands, namely N4,N4′‐bis(pyridin‐2‐ylmethyl)‐[2,2′‐bipyridine]‐4,4′‐dicarboxamide (L1) and N4,N4′‐bis(piperidin‐2‐ylmethyl)‐[2,2′‐bipyridine]‐4,4′‐dicarboxamide (L2), have been successfully synthesized, characterized, and Co(II), Cu(II), and Ni(II) complexes of the ligands have been prepared. A single crystal X‐ray diffraction technique also characterized the L1 structure. The antibacterial activities of the ligands and metal complexes were assessed against resistant strains. The findings suggest that compound L2‐Co exhibits antibacterial properties. Although MIC values were not determined in this study due to the specific concentrations utilized, the results imply that compound L2‐Co holds potential as a promising candidate for further development as an antibacterial agent. Subsequent investigations should prioritize elucidating its mechanism of action and assessing its efficacy in vivo for a comprehensive understanding of its therapeutic potential. The complexes L1‐Co, L1‐Cu, L1‐Ni, L2‐Cu, and L2‐Ni are ineffective against the tested bacteria. There is a need to test higher concentrations to determine effective options. Furthermore, enzyme inhibition studies were conducted, shedding light on the potential therapeutic applications of these complexes in enzyme‐targeted therapies. The L2‐Ni complex showed the strongest inhibition effect for the enzymes studied.

## Conflict of Interest

The authors declare no conflict of interest.

## Data Availability

The data that support the findings of this study are available in the supplementary material of this article.
